# *DNM1* encephalopathy: A new disease of vesicle fission

**DOI:** 10.1212/WNL.0000000000004152

**Published:** 2017-07-25

**Authors:** Sarah von Spiczak, Katherine L. Helbig, Deepali N. Shinde, Robert Huether, Manuela Pendziwiat, Charles Lourenço, Mark E. Nunes, Dean P. Sarco, Richard A. Kaplan, Dennis J. Dlugos, Heidi Kirsch, Anne Slavotinek, Maria R. Cilio, Mackenzie C. Cervenka, Julie S. Cohen, Rebecca McClellan, Ali Fatemi, Amy Yuen, Yoshimi Sagawa, Rebecca Littlejohn, Scott D. McLean, Laura Hernandez-Hernandez, Bridget Maher, Rikke S. Møller, Elizabeth Palmer, John A. Lawson, Colleen A. Campbell, Charuta N. Joshi, Diana L. Kolbe, Georgie Hollingsworth, Bernd A. Neubauer, Hiltrud Muhle, Ulrich Stephani, Ingrid E. Scheffer, Sérgio D.J. Pena, Sanjay M. Sisodiya, Ingo Helbig, For the Epi4K Consortium, For the EuroEPINOMICS-RES NLES Working Group

**Affiliations:** Author affiliations are provided at the end of the article.

## Abstract

**Objective:**

To evaluate the phenotypic spectrum caused by mutations in dynamin 1 (*DNM1*), encoding the presynaptic protein DNM1, and to investigate possible genotype-phenotype correlations and predicted functional consequences based on structural modeling.

**Methods:**

We reviewed phenotypic data of 21 patients (7 previously published) with *DNM1* mutations. We compared mutation data to known functional data and undertook biomolecular modeling to assess the effect of the mutations on protein function.

**Results:**

We identified 19 patients with de novo mutations in *DNM1* and a sibling pair who had an inherited mutation from a mosaic parent. Seven patients (33.3%) carried the recurrent p.Arg237Trp mutation. A common phenotype emerged that included severe to profound intellectual disability and muscular hypotonia in all patients and an epilepsy characterized by infantile spasms in 16 of 21 patients, frequently evolving into Lennox-Gastaut syndrome. Two patients had profound global developmental delay without seizures. In addition, we describe a single patient with normal development before the onset of a catastrophic epilepsy, consistent with febrile infection-related epilepsy syndrome at 4 years. All mutations cluster within the GTPase or middle domains, and structural modeling and existing functional data suggest a dominant-negative effect on DMN1 function.

**Conclusions:**

The phenotypic spectrum of *DNM1*-related encephalopathy is relatively homogeneous, in contrast to many other genetic epilepsies. Up to one-third of patients carry the recurrent p.Arg237Trp variant, which is now one of the most common recurrent variants in epileptic encephalopathies identified to date. Given the predicted dominant-negative mechanism of this mutation, this variant presents a prime target for therapeutic intervention.

Dynamin 1 (*DNM1*; NM_004408) is located on chromosome 9q34.11 and encodes DNM1, a GTPase involved in synaptic vesicle fission for receptor-mediated endocytosis on the presynaptic plasma membrane.^1^ DNM1 self-assembles into multimeric spirals around the necks of budding vesicles.^2^ Functional consequences of *Dnm1* mutations have been demonstrated in animal models such as *Drosophila* temperature-sensitive *shibire* flies, in which mutations caused depletion of synaptic vesicles due to blocked endocytosis.^3,4^ Furthermore, the *fitful* mouse, heterozygous for a spontaneous mutation in the middle domain of *Dnm1*, has recurrent seizures.^5^

De novo mutations in *DNM1* have been identified in patients with severe childhood epilepsies in large-scale genetic studies.^6,7^ Pathogenic variants in *DNM1* account for up to 2% of patients with infantile spasms or Lennox-Gastaut syndrome.^7^

Here, we aim to characterize the phenotypic and genetic spectrum of *DNM1* encephalopathy. We find that patients with *DNM1* encephalopathy have a relatively homogeneous phenotype of severe to profound intellectual disability, hypotonia, and epilepsy starting with infantile spasms with frequent evolution to Lennox-Gastaut syndrome. All de novo mutations cluster in the GTPase and middle domains, and structural modeling provides additional evidence of a dominant-negative mechanism leading to impaired synaptic vesicle endocytosis.

## METHODS

### Patients.

Patients with pathogenic *DNM1* variants were identified between the beginning of 2015 and summer 2016 from various genetic sequencing projects for patients with epilepsy, including the EuroEPINOMICS RES project, Epilepsy Phenome/Genome (EPGP), and Epi4K projects^7^; from patient reports^8,9^; and from diagnostic laboratories. A detailed medical history, including epilepsy, development, and neurologic status, was obtained for each patient. EEG and imaging data were reviewed. Seizures and epilepsy syndromes were classified according to the International League Against Epilepsy (ILAE) classification scheme.^10^

### Standard protocol approvals, registrations, and patient consents.

For patients recruited within the EuroEPINOMICS-RES project or the EPGP/Epi4K project, site-specific institutional review boards approved the study. Patients identified by routine clinical genetic testing gave informed consent according to ethics and legal regulations at the individual centers. Signed informed consent was obtained from all study participants or their legal representatives.

### Mutation analysis.

Mutations were identified with research or clinical testing using next-generation sequencing: patients 3 and 8 from EuroEPINOMICS^7^; patients 5, 7, and 19 from EPGP/Epi4K^6^; and patients 1, 2, 15, 17, and 21 by diagnostic whole-exome sequencing or research protocols. Sequencing and data analysis were performed as previously described.^6,7,11^ Sanger sequencing was used in all patients and parents to confirm the mutation and to study the inheritance of the mutation. None of the patients included in this project were found to have additional explanatory genetic findings.

### Computational structural modeling.

Structures for DNM1 (PDB: 3ZVR),^12^ GDP-AlF4 and magnesium bound to GTPase domain (PDB: 2X2E),^12^ and DNM3 (PDB:5A3F)^13^ were downloaded from the protein data bank.^14^ Graphics were generated with open-source PyMol (www.pymol.org). Variants from the Exome Aggregation Consortium (ExAC) database included only missense changes with ≥2 alleles.^15^

## RESULTS

### Mutational spectrum.

The current study reviewed data from 21 patients, including 19 sporadic patients and a sibling pair (patients 12 and 13, previously reported^9^), resulting in a total of 20 independent mutations (tables 1 and 2). Nine of 20 independent patients (45%) carried recurrent mutations within the *DNM1* gene. The most common mutation was the c.709C>T (p.Arg237Trp) mutation, which was found in 6 of 20 independent patients (30%). In addition, mutations affecting the p.Gly359 amino acid residue were identified in 3 of 20 patients (15%; p.Gly359Ala in 1 patient, p.Gly359Arg in 2 patients), and alterations affecting the p.Lys206 amino acid residue were identified in 2 of 20 patients (10%; p.Lys206Asn and p.Lys206Glu). All mutations were confirmed to be de novo, except for the affected sibling pair whose father was shown to have 5.5% mosaicism on leucocyte DNA.^9^ Fourteen of 20 mutations (70%), including the recurrent c.709C>T (p.Arg237Trp) mutation, occurred in the GTPase domain of *DNM1*.

**Table 1:**
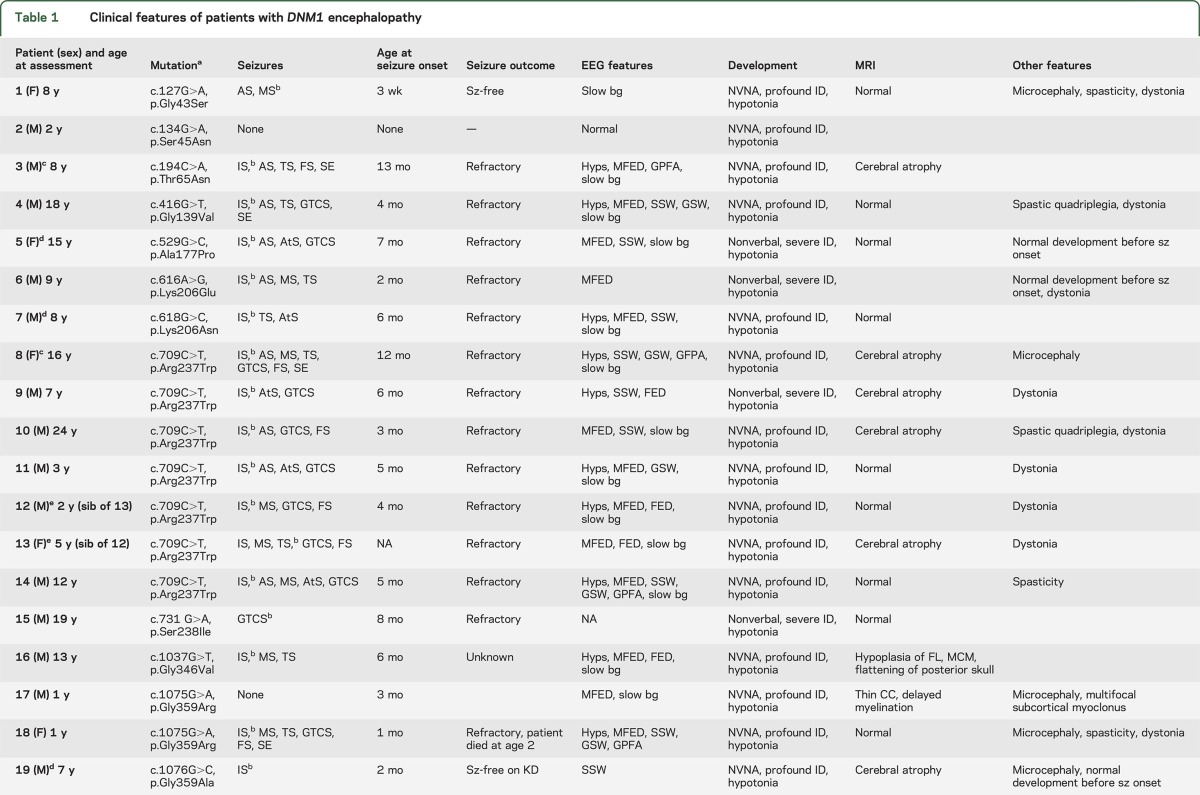

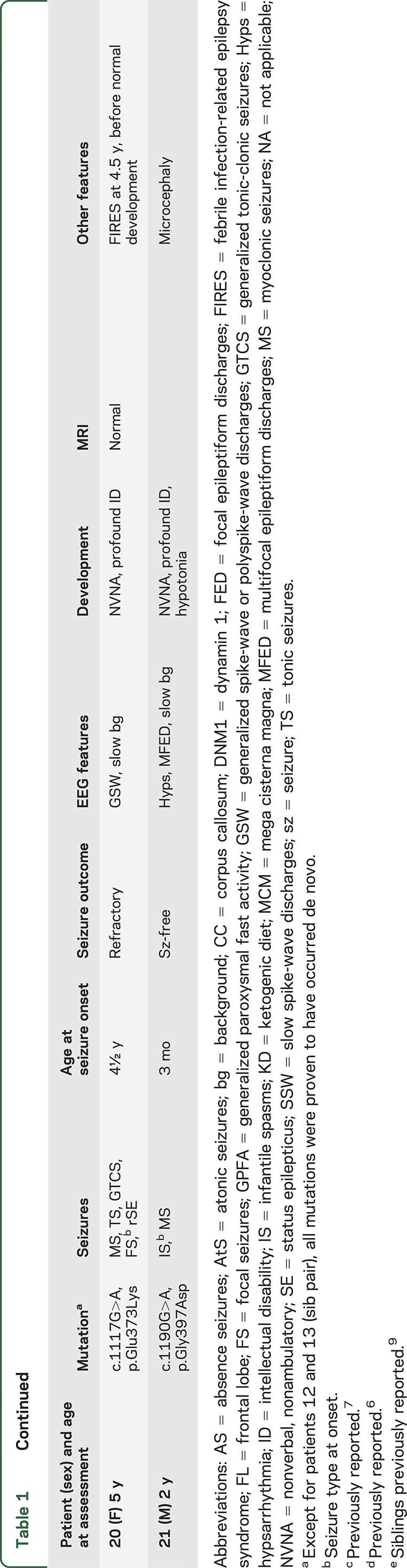
Clinical features of patients with *DNM1* encephalopathy

**Table 2:**
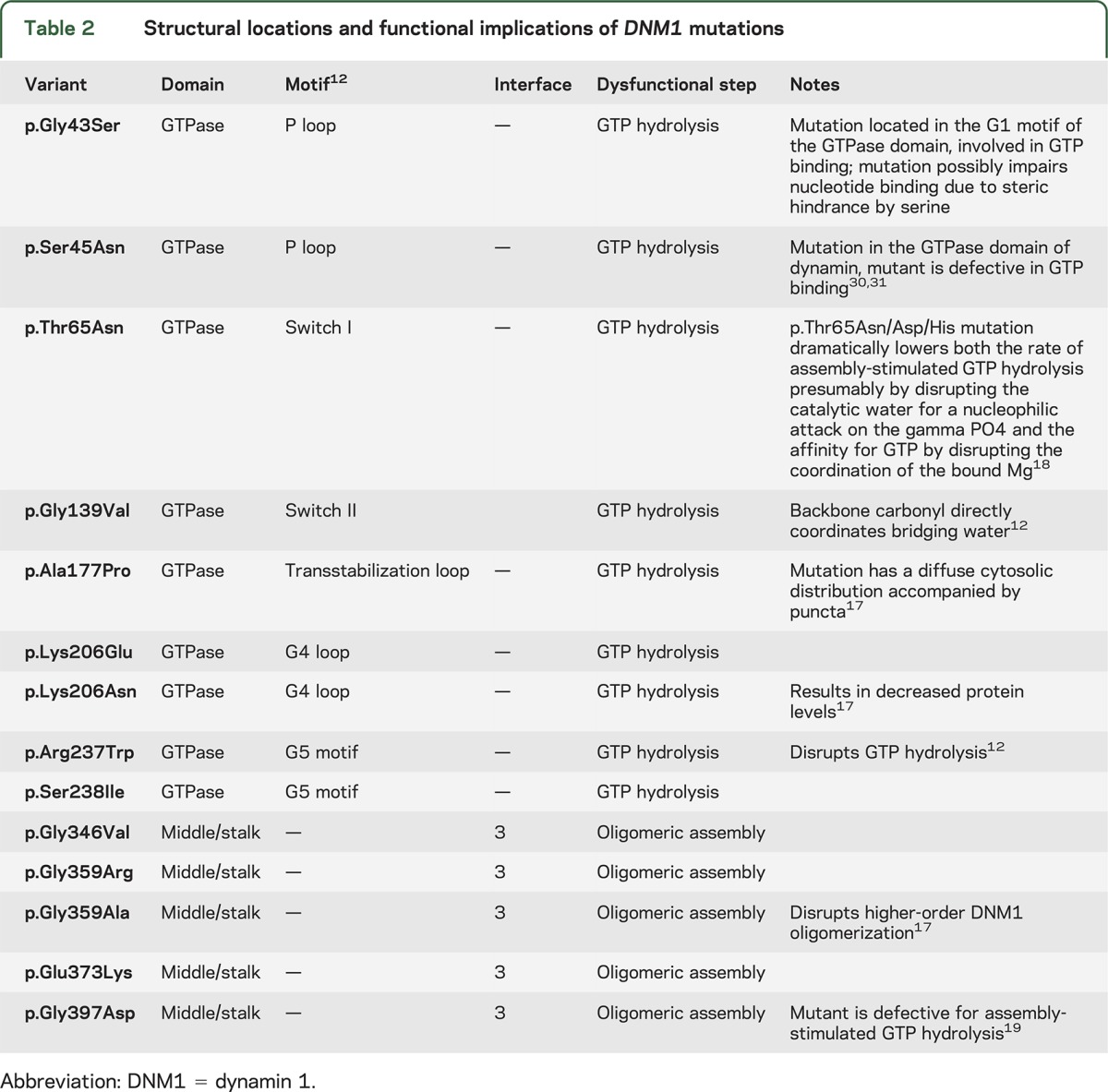
Structural locations and functional implications of *DNM1* mutations

**Figure 1:**
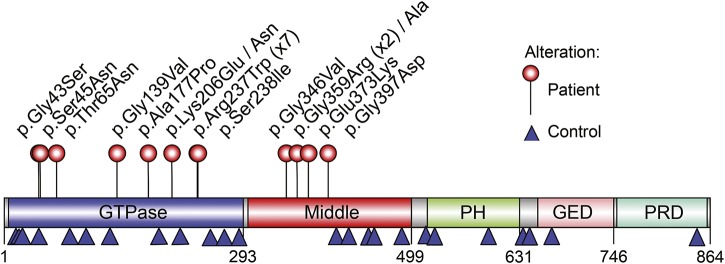
Locations of identified variants in DNM1 protein DNM1 protein (NP_004399) domain structure with locations of variants identified in patients (listed above the image) and controls from population databases such as ESP, ExAC, and 1000Genomes (listed below the image). All members of the dynamin family contain the GTPase domain involved in GTP binding and hydrolysis, the middle domain and GED required for oligomerization and stimulation of the GTPase activity, the PH domain for lipid binding, and the PRD, which interacts with Src-homology-3 domain–containing proteins (figure generated with IBS^32^). DNM1 = dynamin 1; ESP = Exome Sequencing Project; ExAC = Exome Aggregation Consortium; GED = GTPase effector domain; PH = pleckstrin-homology; PRD = proline-rich domain.

**Figure 2:**
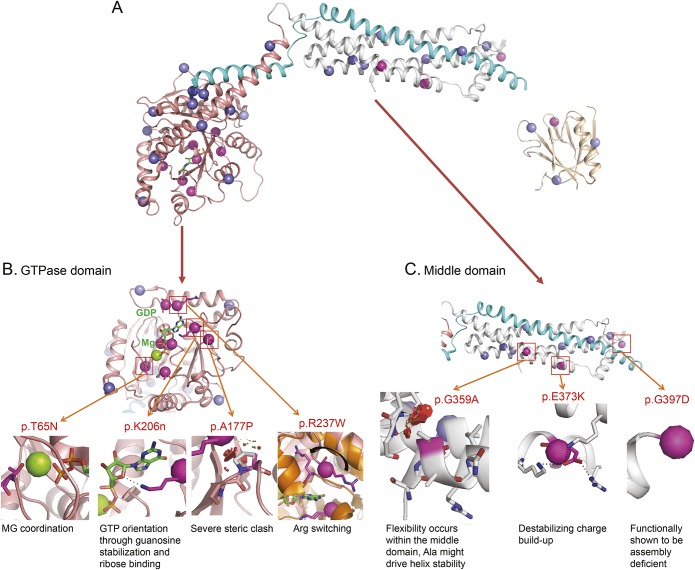
Functional consequences of *DNM1* mutations (A) The entire DNM1 monomer is shown as a cartoon and colored by the GTPase domain (peach), middle domain (white), GED (teal), and PH domain (gold). ExAC missense alteration (blue spheres) and variants discussed in this study (magenta spheres) are shown. (B) Close-up view of the GTPase domain GTP-binding surface with detailed descriptions of the mode of anticipated protein disruption from select observed variants (p.Thr65Asn, p.Ala177Pro, p.Lys206Asn, p.Arg237Trp). (C) Detailed description of the mode of anticipated protein disruption of observed middle domain variants (p.Gly359Ala and p.Gly397Asp). DNM1 = dynamin 1; ExAC = Exome Aggregation Consortium; GED = GTPase effector domain; PH = pleckstrin-homology.

The ExAC dataset^15^ is derived from a control population, and observed variants are considered benign. Plotting the location of missense variants in DNM1 shows a stark pattern of segregation between pathogenic DNM1 variants and benign variants (figures 1 and 2A). The ExAC variants cluster on the surface of the protein with none observed in either the GTP binding pocket of the GTPase domain or any of the oligomerization interfaces in the middle domain. This clustering indicates hot-spot pathogenic regions within the protein.

### Phenotypes of patients with *DNM1* encephalopathy.

For the analysis of *DNM1*-related phenotypes, all 21 patients were included (6 female, 15 male, median age at inclusion 8 years, range 1–24 years). Pregnancy and delivery were unremarkable in all patients with normal birth parameters. Patient 18 died at 2 years of age, before inclusion in this study.

#### Development.

All patients with *DNM1* encephalopathy were nonverbal with severe to profound intellectual disability. In 17 of 21 patients (81%), developmental delay was apparent before seizure onset, while regression started with the onset of seizures in 4 of 21 patients (19%). Except for a single patient (patient 20) who had normal development until the onset of refractory seizures at the age of 4.5 years, all patients had significant developmental delay in the first year of life. Seventeen of 21 patients (81%) were nonambulatory.

#### Seizures.

Seizures occurred in 19 of 21 patients (90%). Patient 2 did not have seizures, and patient 17 showed only subcortical, nonepileptic myoclonic jerks. Seizures began at a median age of 7.6 months (range 1 month–4.5 years). Patient 20 was an outlier with onset at 4.5 years with a febrile infection-related epilepsy syndrome phenotype. In terms of seizure type, 15 of 19 patients presented with infantile spasms, whereas 1 patient each presented with myoclonic seizures, tonic seizures, generalized tonic-clonic seizures (GTCS), and focal seizures; information was not available for 1 patient. Later, spasms occurred in 16 of 19 patients (84%), GTCS in 12 of 19 patients (63%), typical and atypical absence seizures and tonic seizures in 9 of 19 patients (47%), focal seizures in 7 of 19 patients (37%), and atonic seizures in 5 of 19 patients (26%). Five patients had status epilepticus (26%). There were no consistent precipitating factors for seizures: patients 10, 14, and 18 showed sensitivity to higher temperatures and fever; patient 8 had reflex seizures; and patient 17 had nonepileptic myoclonic jerks that were elicited by touch and sound.

#### EEG features.

Abnormal EEG findings were found in all patients with seizures and the patient with nonepileptic myoclonic jerks. One patient (patient 1) had only nonspecific background slowing, while 19 of 20 patients (95%) had epileptiform discharges on EEG and background slowing. Multifocal epileptiform discharges were the most frequent finding, present in 14 of 20 patients (70%). Hypsarrhythmia occurred in 11 of 20 patients (55%). Other epileptiform features observed in a subset of patients included slow spike-wave discharges (9 of 20 patients, 45%), generalized spike-wave activity (6 of 20 patients, 30%), paroxysmal fast activity (4 of 20 patients, 20%), and focal epileptiform discharges (4 of 20 patients, 20%).

#### Medication response.

Seizure outcome was assessed in 18 patients: 15 of 18 patients (83%) had refractory seizures. Three patients (17%) became seizure-free on treatment. Patient 19 became seizure-free on the ketogenic diet (KD) at the age of 3.5 years; patient 21 had a dramatic reduction of myoclonic seizures in the second year of life while on the KD and continues to have frequent nonepileptic myoclonic jerks. Seizures in patient 1 were refractory until the age of 6 years, when she became seizure-free on levetiracetam and lamotrigine.

Improvement in seizure control, but not seizure freedom, was observed with clobazam or clonazepam in 5 of 20 patients (25%), steroids or adrenocorticotropic hormone in 4 of 20 patients (20%), topiramate or zonisamide in 3 of 20 patients (15%), and the KD in 5 of 20 patients (25%). Worsening of seizures was reported in individual patients with levetiracetam, the KD, and cannabidiol. Nonepileptic myoclonic jerks in patient 17 improved on clonazepam.

#### Tone and movement disorders.

A broad range of other neurologic symptoms was observed, including hypotonia in 19 of 21 patients (90%); movement disorders, including choreoathetosis and dystonia, in 11 of 21 patients (52%); and spasticity in 5 of 21 patients (24%). Video recordings of the siblings 2.5 and 4.7 years of age showing their movement disorder and profound hypotonia are available (videos 1–3 at Neurology.org).

#### Dysmorphic features.

Mild dysmorphic features were seen in 8 of 21 patients (38%), but a consistent dysmorphic pattern was not identified. Bitemporal narrowing was found in 3 patients (patients 10, 14, and 17). Eight of 21 patients had microcephaly.

#### Neuroimaging findings.

Brain MRI was performed in 19 of 21 patients (90%) and was unremarkable in 10 of 19 patients (53%). Six of 19 patients (32%) had cerebral volume loss over time, which was observed as early as 2 years (patient 10). Patient 19 had delayed myelination and a thin corpus callosum.

#### Atypical phenotypes.

Our cohort included 3 patients with phenotypes that stood out from the overall group. Patient 2 (c.134G>A; p.Ser45Asn) and patient 17 (c.1075G>A; p.Gly359Arg) did not have epileptic seizures. Patient 2 was studied at 24 months, an age when 18 of 19 patients had already had seizures. He had profound developmental delay and was nonverbal and nonambulatory; his EEG was normal. Patient 17 had profound developmental delay at 1 year. He had multifocal, low-amplitude myoclonic jerks that were shown to be nonepileptic on several EEG recordings. His EEG showed background slowing and multifocal spikes.

Patient 20 (c.1117G>A; p.Glu373Lys) had normal development before the onset of refractory status epilepticus after a mild febrile illness at the age of 4.5 years, a phenotype consistent with febrile infection-related epilepsy syndrome.^16^

#### Phenotype associated with p.Arg237Trp mutation.

Seven of 21 patients (33%) had the recurrent p.Arg237Trp mutation and had infantile spasms with developmental delay before seizure onset, progressing to refractory epilepsy with GTCS. Five of 7 patients (71%) had prominent hyperkinetic movements, dystonic posturing of the head and limbs, and/or ataxic gait in patients who achieved independent ambulation. These clinical features were less frequent in the remainder of the patient cohort, with infantile spasms in 9 of 13 patients (69%), GTCS in 5 of 13 patients (38%), refractory epilepsy in 8 of 11 patients (72%), and movement disorder in 6 of 13 patients (46%). Taken together, the patients with the p.Arg237Trp mutation showed a relatively homogeneous phenotype compared with the overall patient cohort.

#### Adult and older adolescent phenotype.

Four patients were ≥16 years of age (patients 4, 8, 10, and 15); 2 had the recurrent p.Arg237Trp mutation (patients 8 and 10). All had multidrug-resistant epilepsy persisting into adulthood, and their phenotype did not differ from that of the overall cohort. One patient developed severe reflex seizures triggered by actions of personal hygiene such as brushing her teeth. All patients had severe intellectual disability; only one patient was ambulatory.

### Computational structural modeling.

All GTPase domain variants occur in key catalytic motifs (p.Gly43Ser, p.Ser45Asn, p.Thr65Asn, p.Gly139Val, p.Ala177Pro, p.Lys206Asn, p.Lys206Glu, p.Arg237Trp, and p.Ser238Ile), and all affect GTP binding, hydrolysis, or stability (figure 2B). Two of the observed variants (p.Thr65Asn and p.Arg237Trp) have been previously described.^12^ Briefly, the position p.Thr65 is present in the switch I motif and coordinates a bound Mg^2+^ where substitution for an asparagine (p.Thr65Asn) would greatly impair GTP hydrolysis (figure 2B). The p.Gly139Val variant is present in the switch II motif that positions a bridging water in the catalytic site adjacent to the Thr65 position.^12^ The p.Arg237Trp variant occurs in the dynamin-specific G5 motif. The p.Arg237 residue stabilizes the transition state by undergoing a 180° rotation during GTP hydrolysis, causing a large conformational switch in the surrounding region.^12^ This switching is likely to be perturbed by the larger tryptophan residue (figure 2B).

The p.Lys206Asn and p.Lys206Glu variants occur in the nucleotide specificity G4 loop of the GTPase domain and interact with the guanosine and ribose moieties of the bound GTP (figure 2B).^12^ This position is involved in direct contact with the bound nucleotide and likely influences its stabilization and binding. The p.Lys206Asn variant leads to a decrease in protein stability,^17^ suggesting GTP binding as a stabilization step.

In addition, 2 other variants (p.Gly43Ser and p.Ser45Asn) result in GTP affinity perturbations. Both interact via the oxygen/phosphate backbone with the bound GDP. Point mutations at position 45 are defective in GTP binding.^18^

The p.Ala177Pro variant, found in the transstabilizing loop, introduces a severe steric clash near the p.Arg237 switching residue. The proline introduces more steric constraint and affects the dynamics of the arginine in position 237 (figure 2B). The p.Ser238Ile variant results in a disruption of p.Arg237 switching dynamics (figure 2B). The p.Ser238 residue rotates from a buried position to a solvent-exposed position that is sandwiched between the bound nucleotide cofactor and acts to stabilize the helix dipole formed by the p.Arg237 movement. The variant p.Ser238Ile introduces an energetically unfavorable hydrophobic residue. This results in a perturbation to the DNM1 p.Arg237 switching mechanism.

Four variants are localized to the middle domain of DNM1 (p.Glu373Lys, p.Gly359Ala, p.Gly359Arg, and p.Gly397Asp) (figure 2C). They occur at the tetramer interface 3 and likely disrupt oligomerization. The p.Gly397Asp variant leads to a buildup of positive charge, disrupting normal interactions. Studies of the crystal structure of rat DNM1 indicate that this mutation interferes with self-assembly, leading to impaired GTP hydrolysis and stalled synaptic endocytosis.^19^

## DISCUSSION

Here, we describe the phenotypic spectrum of patients with de novo mutations in *DNM1*, encoding a key component of synaptic vesicle recycling. *DNM1* initially emerged as a novel disease gene in large-scale genetic studies, but the full phenotypic and genetic spectrum has not yet been described. We show that patients with *DNM1* mutations have a phenotype characterized by intellectual disability, hypotonia, and refractory epilepsy typically presenting with infantile spasms. One-third of patients had the recurrent c.709C>T (p.Arg237Trp) mutation and a homogeneous phenotype.

Mutations in genes encoding synaptic vesicle proteins are increasingly recognized as causal for neurodevelopmental disorders, including the epilepsies. Implicated synaptic genes include *DNM1, STXBP1*, *STX1B*, and *SNAP25*.^20–22^ The DNM1 protein is a key component of vesicle recycling. It is the main driver for invagination of clathrin-coated synaptic vesicles, which occurs through a process of GTP-mediated oligomeric assembly.^23,24^ The predominance of intractable epilepsies in patients with *DNM1* encephalopathy reinforces the notion that in addition to disrupted synaptic vesicle fusion, disordered vesicle recycling may represent a key step in epileptogenesis.

*DNM1* encephalopathy is a disease of vesicle fission, and the mutations in our patient cohort cluster in 2 major functional domains of the DNM1 protein: the GTPase domain and the middle domain. Our structural modeling studies suggest a dominant-negative effect for all mutations studied. Even though all mutations are predicted to impair the process of endocytosis, the locations of the variants indicate disruptions at different stages of vesical invagination (figure 2). Most of the variants in the GTPase domain are predicted to impair GTP hydrolysis but not the binding to the synaptic vesicle, resulting in intact oligomeric assembly but impaired vesicle scission,^24^ which has been shown experimentally for the p.Ala177Pro variant.^17^ In contrast, middle domain variants are predicted to affect the ability of DNM1 to form larger oligomeric assemblies. Accordingly, even though mutations in both domains result in comparable phenotypes, we predict that the mechanism of protein disruption is different.

The relative phenotypic homogeneity of patients with *DNM1* mutations is remarkable and might guide clinicians to initiate targeted genetic testing especially when gene panel analysis or exome sequencing is not available. While mutations in other well-recognized epilepsy-causing genes such as *SCN2A*, *SCN8A*, or *STXBP1* result in a wide range of disorders, including mild and severe epilepsies and even isolated autism spectrum disorders,^20,25,26^ the majority of patients with *DNM1* encephalopathy have a relatively homogeneous developmental and epileptic encephalopathy. The onset of seizures after the neonatal period may be explained by the expression of the DNM1 protein. *DNM1* expression increases postnatally in parallel to synapse formation; however, DNM1 is not required for initial synapse formation.^27,28^

Patients with *DNM1* encephalopathy typically have intractable epilepsy with limited efficacy of antiepileptic medications. While the KD and treatment with benzodiazepines provided benefit in some patients, most patients had intractable epilepsy that continued into adulthood. This contrasts with many genetic epilepsies such as *SCN2A*, *KCNQ2*, and *STXBP1* encephalopathy, in which some patients show seizure remission despite severe long-term developmental consequences. However, during the initial course of the epilepsy, patients with various genetic causes can have remarkably similar clinical presentations.

Patients with the recurrent p.Arg237Trp mutation account for one-third of patients with *DNM1* encephalopathy in our series. Given that *DNM1* mutations account for up to 2% of patients with severe epilepsy,^7^ this mutation may represent one of the most frequent single mutations in patients with epileptic encephalopathies. The relatively homogeneous phenotype and the predicted dominant-negative mechanism of this mutation make *DNM1* encephalopathy an interesting therapeutic target for pharmacologic approaches and gene therapy to restore DNM1 function.

Our cohort of 21 cases is relatively small because *DNM1* encephalopathy has only recently been discovered.^6,7^ Recruitment bias may have distorted the phenotypic picture of patients with *DNM1* encephalopathy because patients have been recruited largely through studies of epileptic encephalopathies. However, we have reason to believe that the phenotypic spectrum with prominent epilepsy presented in our study reflects the overall clinical picture of *DNM1* encephalopathy. First, we included patients ascertained from a large diagnostic laboratory, which is a source of relatively unbiased recruitment with regard to the epilepsy phenotype. Second, we reviewed the available data in the Deciphering Developmental Disorders Study.^29^ In this cohort, 5 patients were identified with nonsynonymous de novo missense mutations in a cohort of patients with broad developmental disorders. Human phenotype ontology terms of these patients indicate that 4 of the 5 patients had seizures, suggesting that epilepsy is a prominent feature of *DNM1* encephalopathy. In addition, our cohort comprises twice as many male as female patients. Whether this reflects a true preponderance of male patients or is simply due to chance remains unclear at the moment.

We delineate the phenotypic spectrum of *DNM1* encephalopathy, an emerging disease of synaptic vesicle fission. This developmental and epileptic encephalopathy is characterized by severe to profound developmental delay, infantile-onset epilepsy beginning with infantile spasms, and movement disorder. The genetic landscape of *DNM1* encephalopathy is notable for the recurrent c.709C>T (p.Arg237Trp) variant, together with localization of mutations to specific domains of the protein. Characterizing *DNM1* encephalopathy as a unique condition leading to an intractable epileptic encephalopathy will lay the foundation for the development of targeted therapies.

## AUTHOR AFFILIATIONS

From the Department of Neuropediatrics (S.v.S., M.P., H.M., U.S.), University Medical Center Schleswig-Holstein, Kiel; Northern German Epilepsy Center for Children and Adolescents (S.v.S.), Schwentinental, Germany; Division of Clinical Genomics (K.L.H., D.N.S.) and Department of Bioinformatics (R.H.), Ambry Genetics, Aliso Viejo, CA; Neurogenetics Unit (C.L.), Clinics Hospital of Ribeirao Preto, Sao Paulo, Brazil; Department of Genetics (M.E.N., R.A.K.), Kaiser Permanente, San Diego; Department of Neurology (D.P.S.), Kaiser Permanente, Los Angeles, CA; Division of Neurology (D.J.D., I.H.), The Children's Hospital of Philadelphia, PA; Department of Neurology and Pediatrics (H.K., M.R.C.) and Department of Pediatrics (A.S.), Division of Genetics, University of California, San Francisco; Department of Neurology (M.C.C.), Johns Hopkins Hospital; Division of Neurogenetics (J.S.C., R.M., A.F.), Hugo W. Moser Research Institute, Kennedy Krieger Institute, Baltimore, MD; Genomics Institute (A.Y.), Multicare Tacoma General Hospital, WA; Division of Child Neurology (Y.S.), Children's Hospital of Pittsburgh of UPMC, PA; Children's Hospital of San Antonio (R.L., S.D.M.), Baylor College of Medicine, TX; Institute of Neurology (L.H.-H., B.M., S.M.S.), UCL, London; Epilepsy Society (L.H.-H., B.M., S.M.S.), Bucks, UK; Danish Epilepsy Centre (R.S.M.), Dianalund; Institute for Regional Health Services (R.S.M.), University of Southern Denmark, Odense; Departments of Medical Genetics (E.P.) and Neurology (J.A.L.), Sydney Children's Hospital, Randwick; Genetics of Learning Disability Service (E.P.), Royal North Shore Hospital, St Leonards, Sydney, NSW, Australia; Iowa Institute of Human Genetics (C.A.C., D.L.K.) and Department of Internal Medicine (C.A.C.), University of Iowa, Carver College of Medicine; Division of Pediatric Neurology (C.N.J.), University of Iowa, Children's Hospital, Iowa City; Children's Hospital Colorado (C.N.J.), Colorado Springs; Epilepsy Research Centre (G.H., I.E.S.), Department of Medicine, Austin Health, University of Melbourne, Heidelberg; Department of Paediatrics (I.E.S.), University of Melbourne, Royal Children's Hospital, Parkville; Florey Institute of Neurosciences and Mental Health (I.E.S.), Melbourne, Victoria, Australia; Department of Neuropediatrics (B.A.N.), University Hospital of Giessen and Marburg, Giessen, Germany; and GENE–Núcleo de Genética Médica (S.D.J.P.), Belo Horizonte, Brazil.

## GLOSSARY

DNM1: dynamin 1
EPGP: Epilepsy Phenome/Genome
ExAC: Exome Aggregation Consortium
GTCS: generalized tonic-clonic seizures
ILAE: International League Against Epilepsy
KD: ketogenic diet
